# HIV gp41 Engages gC1qR on CD4+ T Cells to Induce the Expression of an NK Ligand through the PIP3/H2O2 Pathway

**DOI:** 10.1371/journal.ppat.1000975

**Published:** 2010-07-01

**Authors:** Hugues Fausther-Bovendo, Vincent Vieillard, Sandrine Sagan, Georges Bismuth, Patrice Debré

**Affiliations:** 1 Laboratoire Immunité et Infection, Institut National de la Santé et de la Recherche Médicale UMR-S 945 I, Paris, France; 2 Université Pierre et Marie Curie (Paris 6), Paris, France; 3 Laboratoire des Biomolécules, Centre National de la Recherche Scientifique UMR 7203, Paris, France; 4 Institut Cochin, Université Paris Descarte, Centre National de la Recherche Scientifique UMR 8104, Equipe labellisée par la Ligue Nationale contre le Cancer, Paris, France; 5 Institut National de la Santé et de la Recherche Médicale, U567, Paris, France; Northwestern University, United States of America

## Abstract

CD4^+^ T cell loss is central to HIV pathogenesis. In the initial weeks post-infection, the great majority of dying cells are uninfected CD4^+^ T cells. We previously showed that the 3S motif of HIV-1 gp41 induces surface expression of NKp44L, a cellular ligand for an activating NK receptor, on uninfected bystander CD4^+^ T cells, rendering them susceptible to autologous NK killing. However, the mechanism of the 3S mediated NKp44L surface expression on CD4^+^ T cells remains unknown. Here, using immunoprecipitation, ELISA and blocking antibodies, we demonstrate that the 3S motif of HIV-1 gp41 binds to gC1qR on CD4^+^ T cells. We also show that the 3S peptide and two endogenous gC1qR ligands, C1q and HK, each trigger the translocation of pre-existing NKp44L molecules through a signaling cascade that involves sequential activation of PI3K, NADPH oxidase and p190 RhoGAP, and TC10 inactivation. The involvement of PI3K and NADPH oxidase derives from 2D PAGE experiments and the use of PIP3 and H2O2 as well as small molecule inhibitors to respectively induce and inhibit NKp44L surface expression. Using plasmid encoding wild type or mutated form of p190 RhoGAP, we show that 3S mediated NKp44L surface expression on CD4^+^ T cells is dependent on p190 RhoGAP. Finally, the role of TC10 in NKp44L surface induction was demonstrated by measuring Rho protein activity following 3S stimulation and using RNA interference. Thus, our results identify gC1qR as a new receptor of HIV-gp41 and demonstrate the signaling cascade it triggers. These findings identify potential mechanisms that new therapeutic strategies could use to prevent the CD4^+^ T cell depletion during HIV infection and provide further evidence of a detrimental role played by NK cells in CD4^+^ T cell depletion during HIV-1 infection.

## Author Summary

HIV infected individuals suffer from a loss of CD4^+^ lymphocytes. Initially, dying CD4^+^ lymphocytes are mainly infected ones. Afterward, the great majority of dying CD4^+^ lymphocytes are uninfected. The cause of uninfected CD4^+^ lymphocyte death during HIV infection is still under debate. We previously showed that one of the HIV-1 envelop proteins, gp41, induces the expression of a stress molecule called NKp44L on the surface of uninfected CD4^+^ lymphocytes. Uninfected CD4^+^ lymphocytes expressing NKp44L are killed, *in vitro* and *in vivo*, by cells of the immune system called NK cells. In this report, we study the CD4^+^ lymphocyte's proteins involved in the expression of NKp44L. To do so, we used several techniques to identify interacting or differentially expressed proteins and to inhibit or monitor enzymes activity. We also induce NKp44L using the product of some of the proteins involved in NKp44L expression. We found that HIV-1 gp41 binds to its receptor gC1qR on CD4^+^ lymphocytes. This interaction respectively activates the PI3K, the NADPH oxidase and p190 RhoGAP which inactivates TC10. Using the obtained data we build a model of the protein cascade involved in NKp44L surface expression.

## Introduction

CD4 cell depletion and the resulting immune dysfunction are hallmarks of HIV-1 infection. Although this phenomenon appears to be the principal component of HIV disease, its underlying mechanisms remain controversial. The CD4^+^ T cell loss observed during the chronic phase of infection was initially thought to result from the death of productively infected CD4^+^ T cells, due to the cytopathic effects of viral replication and cytotoxic CD8-mediated killing [1], [2]. Several studies have demonstrated, however, that most dying cells are uninfected and thus suggest that the death of uninfected bystander CD4^+^ T cells plays a major role in CD4 depletion [1], [3]. Since then, numerous studies have strengthened this hypothesis. Several HIV-1 proteins, including gp120, Tat, and Nef, activate a variety of disparate pathways to initiate apoptosis in uninfected cells [4]. An alternative proposal is that the high level of immune activation in HIV-infected individuals may cause the killing of uninfected CD4^+^ T cells, by activation-induced cell death (AICD) through Fas [5]. The importance of CD4^+^ T cell preservation in this disease is highlighted by findings in African green monkeys infected with SIVagm: despite high viral replication, their CD4^+^ T cell counts remain normal, and they do not develop AIDS [6].

Increasing evidence suggests that NK cells are involved in CD4^+^ T cell depletion. We have shown that after HIV infection a significant fraction of the CD4-cell subset expresses NKp44L, an activator ligand of the natural cytotoxicity receptor NKp44 [7]. NKp44L expression renders CD4^+^ T cells sensitive to autologous NK lysis. This expression is strongly correlated with both the decline in the CD4 cell count and the increase in viral load [8]. The role of NKp44L in CD4^+^ T cell depletion was confirmed *in vivo* in an SHIV-infected macaque model [9]. Ward *et al.* [*10*] demonstrated that the ligand for NKp44 is expressed only on uninfected bystander CD4^+^ T cells. More recently, we showed that Nef, one of the main effector proteins involved in HIV immune evasion, mediates intracellular retention of NKp44L in HIV-infected CD4^+^ T cells [11]. These results suggest that HIV-1 has acquired the ability to use NK cells to disarm the host immune system by selectively triggering the death of uninfected CD4^+^ T cells.

We have previously showed that a specific motif of HIV-gp41, which is called 3S and is highly conserved in HIV-1 isolates, plays a pivotal role in NKp44L expression [8]. HIV-gp41 protein is a subunit of the Env complex protein and is essential for HIV entry into target cells [12]. The interaction of gp120 with the target cell surface induces conformational changes in the gp120-gp41 complex that expose the gp41 molecules and appear to promote fusion between the viral and cellular membranes [13]. During this process, several gp41 motifs become accessible to the surface, including the 3S motif located between residues 618–623 [8], [14]. Studies in a macaque model have confirmed the important role of this 3S motif in HIV-1 pathogenicity and showed that immunization with the 3S motif prevents NKp44L expression and CD4^+^ T cell depletion following SHIV infection [15].

Interestingly, it has been reported that the HIV-gp41 motif located between residues 601–620 binds to the complement component 1q (C1q) and then activates the classic complement cascade in the absence of antibody [16]. In addition, the gp41 epitope between residues 615–635, which includes the 3S motif, interacts with complement factor H, a negative regulator of the complement cascade that can induce virus opsonization [17], [18]. Its simultaneous triggering of the complement cascade pathway and binding to one of its negative regulators may make HIV-1 gp41 crucial for viral spread and for T cell susceptibility to HIV infection [17], [19].

In this study, we sought to identify the receptor of the HIV-gp41 3S motif on CD4^+^ T cells and examined the signaling pathway leading to NKp44L surface expression. We discovered that the 3S motif binds to gC1qR, a receptor for the globular domain of C1q, on CD4^+^ T cells. This interaction activates successively the PI3K, the NADPH oxidase, and the p190A RhoGAP proteins. Activation of this signaling cascade induces the translocation of pre-existing NKp44L molecules from the cytoplasm to the plasma membrane of CD4^+^ T cells via a TC10-dependent mechanism.

## Results

### The 3S motif of HIV-gp41 stimulates ROS production by NADPH oxidase

We investigated the signaling cascade triggered by the 3S peptide to induce NKp44L cell-surface expression on CD4^+^ T cells. To identify the proteins involved in this cascade, we subjected total protein extracts of U2 cells to a standard two-dimensional polyacrylamide gel electrophoresis (2D PAGE), in the presence or absence of 3S peptide. Proteins differentially expressed by more than twofold were identified by mass spectrometry (MALDI-TOF). Table 1 summarizes in detail the analytic data for each identified spot. The up-regulation of peroxiredoxin and the presence of oxidized forms of GADPH, characterized by their low isoelectric points, indicated the presence of hydrogen peroxide (H_2_0_2_) in cells incubated with the 3S peptide [20].

**Table 1:** List of proteins differentially expressed at least two-fold in U2 cells incubated for 4 h in presence of the 3S peptide.

Name	Mw (KDa)	pI	error (ppm)	score	Expression
Ran specific GTPase activating protein	23.467	5.19	23	81	Underrexpressed
Importin Subunit beta-1	98.42	4.68	41	36	Underrexpressed
Peroxiredoxin	22.3	8.27	42	71	Overexpressed
Heterogenous nuclear ribo-nucleoprotein F	45.99	5.38	29	91	Overexpressed
Mannose-6-phosphate receptor binding protein-1	47.189	5.3	30	141	Overexpressed
GTP binding nuclear protein Ran	24.579	7.01	30	100	Overexpressed
Lisoaspartate (D-aspartate) 0 methyltransferase	24.806	6.7	9	93	Overexpressed
Proteasome subunit alpha Type-2	25.496	6.92	44	127	Overexpressed
calcyclin binding protein+Adenylate kinase isoenzyme -2, mitochondria	26.308, 26.689	8.28, 7.67	7, 5	160	Overexpressed
Spermidine synthase	34	5.3	19	165	Overexpressed
G3PDH+ 60S acidic ribosomal protein 60	36, 34	8.6, 5.6	21, 23	193	Overexpressed
G3PDH	36	8.6	12	80	Overexpressed
IF2A eukaryote translational initiation factor-2	36.4	5.02	16	84	Overexpressed

**Figure 1:**
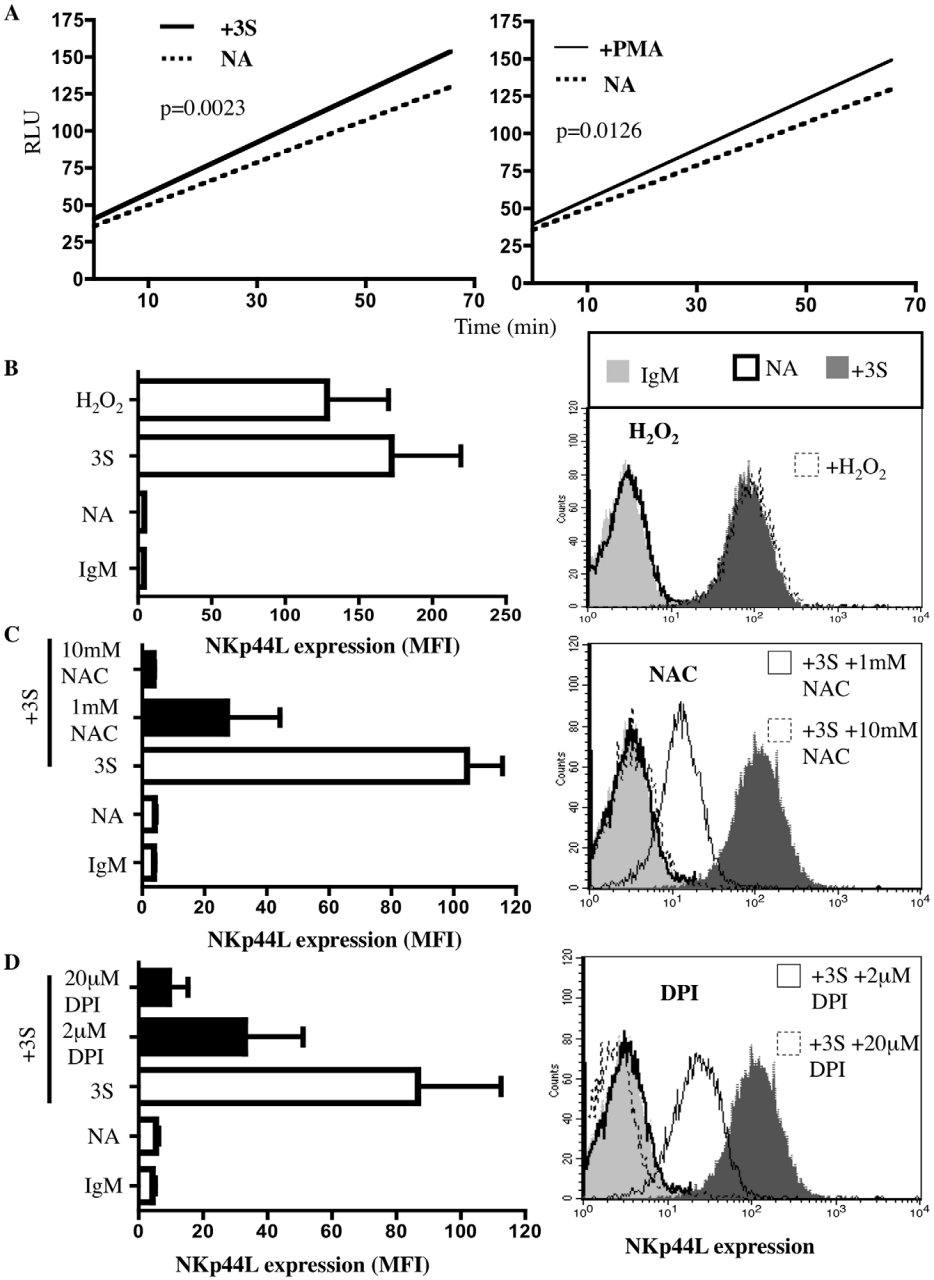
3S peptide stimulation induces ROS production by NADPH oxidase. (A) Intracellular production of reactive oxygen species (ROS) was measured with ROS-sensitive DCFH-DA. CD4^+^ T cells were pre-incubated with 10 µM DCFH-DA. Samples were then incubated in the absence of peptide (dotted line), in the presence of 20 µg/ml 3S peptide (thick solid line) or in the presence of 0.2 µg/ml PMA (thin solid line). DCFH-DA fluorescence was then measured by luminometer every 3 min for 66 min. The curves shown are representative of experiments performed on purified CD4^+^ T cells from 5 individuals. (B) Induction of NKp44L expression by H_2_O_2_. CD4^+^ T cells were stained with IgM isotype control (IgM), or CD4^+^ T cells that were unstimulated (NA), or that were stimulated with 5 µg/ml 3S peptide (3S) or with 100 µM H_2_O_2_ were stained with anti-NKp44L mAb. (C) NAC, an antioxidant, blocks NKp44L surface expression. CD4^+^ T cells were pre-incubated without inhibitor (open bar) and with 1 mM NAC or with 10 mM NAC (black bars), and then stimulated with the 3S peptide. They were then stained with anti-NKp44L mAb. Unstimulated CD4^+^ T cells stained with anti-NKp44L mAb (NA) were used as negative controls. (D) DPI, a NADPH oxidase inhibitor, also blocks NKp44L surface expression. CD4^+^ T cells were pre-incubated without inhibitor (open bar) and with 2 µM DPI, or with 20 µM DPI (black bars), and then stimulated with the 3S peptide, before staining with anti-NKp44L mAb. Unstimulated CD4^+^ T cells stained with anti-NKp44L mAb (NA) were used as negative controls. For (B), (C) and (D) (Left) The mean +/− SD of NKp44L Mean fluorescence intensity (MFI) from experiments using CD4^+^ T cells from 3 individuals are indicated. (Right) One representative experiment is illustrated as FACS histograms.

To confirm the production of H_2_0_2_ during the 3S peptide-mediated signaling, the intracellular concentration of H_2_0_2_ was measured with DCFH-DA in purified CD4^+^ T cells incubated with or without the 3S peptide. Phorbol 12-myristate 13 acetate (PMA), which induces H_2_0_2_ production, was used as a positive control [21]. As Figure 1A shows, incubation with 20 µg/ml 3S peptide induced an increase in the intracellular H_2_0_2_ concentration, similar to the level observed following PMA incubation.

To confirm the role played by H_2_0_2_ in NKp44L surface expression, we incubated CD4^+^ T cells with H_2_0_2_ and detected similar levels of NKp44L surface expression on the CD4^+^ T cells treated with 100 µM H_2_0_2_ and with 5 µg/ml 3S peptide (Figure 1B). Next, assessment of the effect of N-acetyl L cysteine (NAC), an antioxidant, on NKp44L surface expression showed that 10 mM NAC completely abolished this surface expression on purified CD4^+^ T cells treated with 5 µg/ml 3S peptide (Figure 1C). To confirm a previous report that H_2_0_2_ synthesis in lymphocytes depends on NADPH oxidase activation [20], we treated cells with diphenyleneiodonium chloride (DPI), an NADPH oxidase inhibitor, and observed a drastic decrease in 3S-mediated NKp44L cell-surface expression (Figure 1D).

Together, these data indicate that NADPH oxidase may play a key role in the signaling pathway induced by the 3S peptide that leads to NKp44L surface expression and showed that H_2_0_2_ is sufficient to induce this expression.

### The 3S motif of HIV-gp41 activates p190 RhoGAP

**Figure 2:**
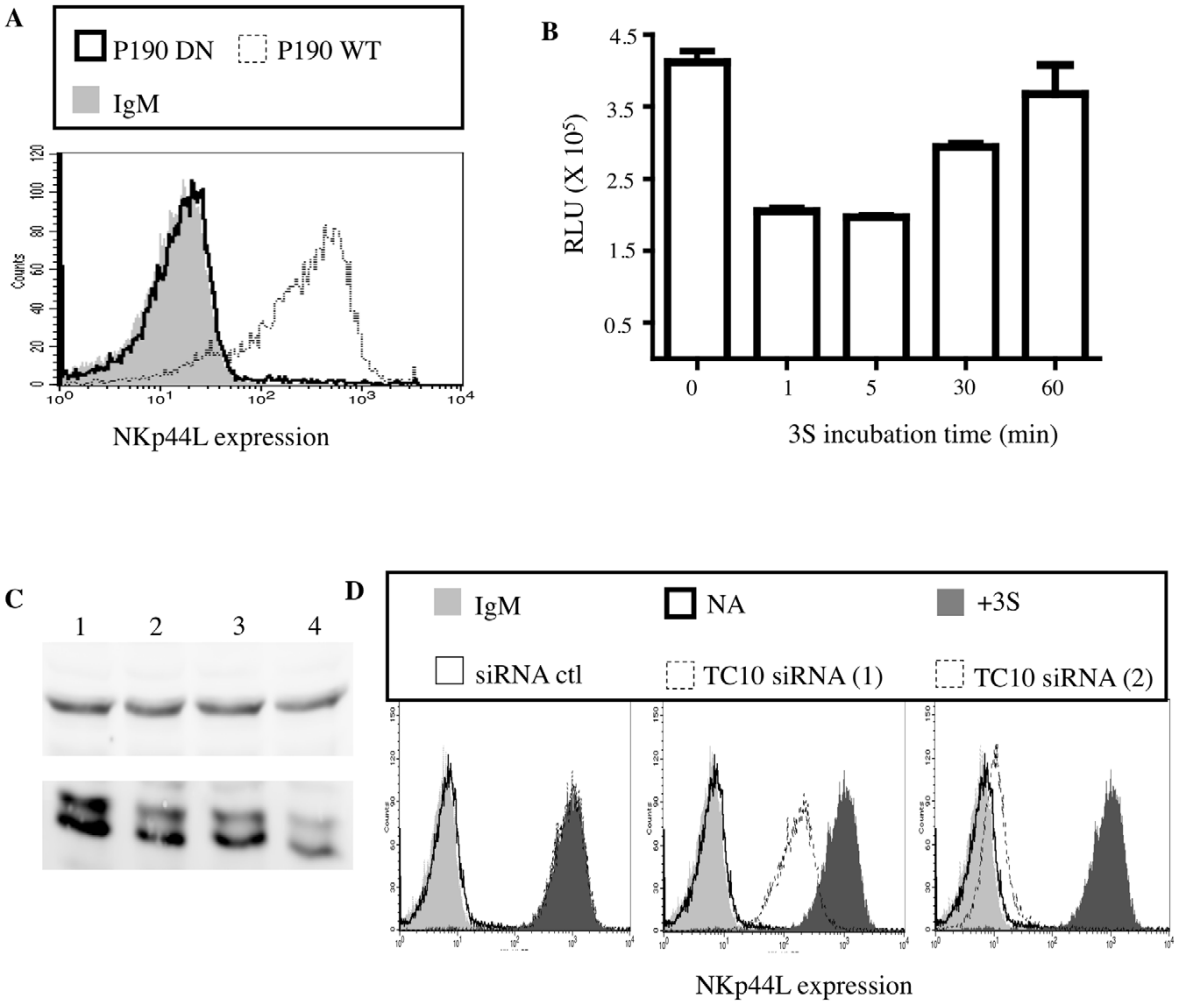
3S peptide stimulation downregulates Rho activity via p190 RhoGAP A. (A) Purified CD4^+^ T cells transfected with deficient p190 RhoGAP A (bold line) or wild-type p190 RhoGAP A (dotted line) were stained with anti-NKp44L antibodies. CD4^+^ T cells stained with IgM isotype control (light gray) were used as the negative control. (B) Rho activity was measured in lysate of CD4^+^ T cells incubated with 5 µg/ml 3S peptide for various lengths of time ranging from 1 to 60 min. (C) Western blot using lysate of CD4+ T cells pre-incubated in absence of siRNA (Lane 1) or with control siRNA (Lane 2) or with siRNA against TC10 (Lane 3 & 4). Both Tubulin (top panel) and TC10 (bottom panel) were monitored. (D) TC10 siRNA inhibits NKp44L cell-surface expression. Unstimulated CD4^+^ T cells stained with IgM isotype control (light gray) or with anti-NKp44L mAb (bold line) were used as negative controls. CD4^+^ T cells were pre-incubated without siRNA (dark gray), with 1 mM control siRNA (thin solid line), 1 mM of either siRNA against TC10 (dotted lines) before stimulation with the 5 µg/ml 3S peptide. Samples were then stained with anti-NKp44L mAb.

Next, we sought to identify the molecules involved downstream of H_2_0_2_ production in 3S-mediated signal transduction. Nimnual *et al.* [22] described the redox-dependent activation of p190 RhoGAP: H_2_0_2_ produced by NADPH oxidase inhibits the low molecular weight protein tyrosine phosphatase (LMW-PTP), which in turn inhibits p190 RhoGAP A (p190A) activity by dephosphorylating it. To determine whether p190A functions as a downstream effector of H_2_0_2_ in NKp44L surface expression, we used purified CD4^+^ T cells transfected with plasmid containing either wild-type p190 RhoGAP A (p190 WT) or its dominant negative form (p190 DN). Remarkably, transient transfection with p190 WT was sufficient to induce NKp44L cell-surface expression, while overexpression of p190 DN had no effect (Figure 2A).

As previously reported, p190A stimulates the GTPase activity of some Rho GTPases, including RhoA and its isoforms RhoB-C as well as TC10, and therefore promotes their GDP-bound form [23], [24]. Accordingly, we used a commercial ELISA-based assay to investigate RhoA activity in the lysate of purified CD4^+^ T cells in the presence of the 3S peptide. As expected, after 3S peptide treatment, RhoA activity decreased transiently by twofold, before returning to its initial level (Figure 2B).

### NKp44L surface expression is TC10-dependent

TC10/RhoQ is a member of the Rho GTPase family and, like other members of this family, it cycles between an inactive GDP-bound state and an active GTP-bound form. Interestingly, TC10 is mainly located on vesicles that are visible throughout the cytoplasm [25]. To determine whether TC10 plays a role in 3S-mediated NKp44L surface expression, TC10 was knocked down by RNA interference (RNAi) in purified CD4^+^ T cells before 3S peptide stimulation. The down-modulation of TC10 in the presence of specific siRNAs was confirmed by western blot (Figure 2C). As Figure 2D shows, two specific TC10 siRNAs strongly reduced NKp44L cell-surface expression after incubation with the 3S peptide, while control siRNA had no effect. Interestingly, the level of NKp44L down-regulation by specific TC10 siRNAs correlated with their ability to down-modulate TC10. This result indicates that TC10 is required for NKp44L cell-surface expression and probably plays a key role in the fusion of vesicles containing NKp44L.

### The 3S motif of HIV-gp41 induces NKp44L translocation to the cell surface

**Figure 3:**
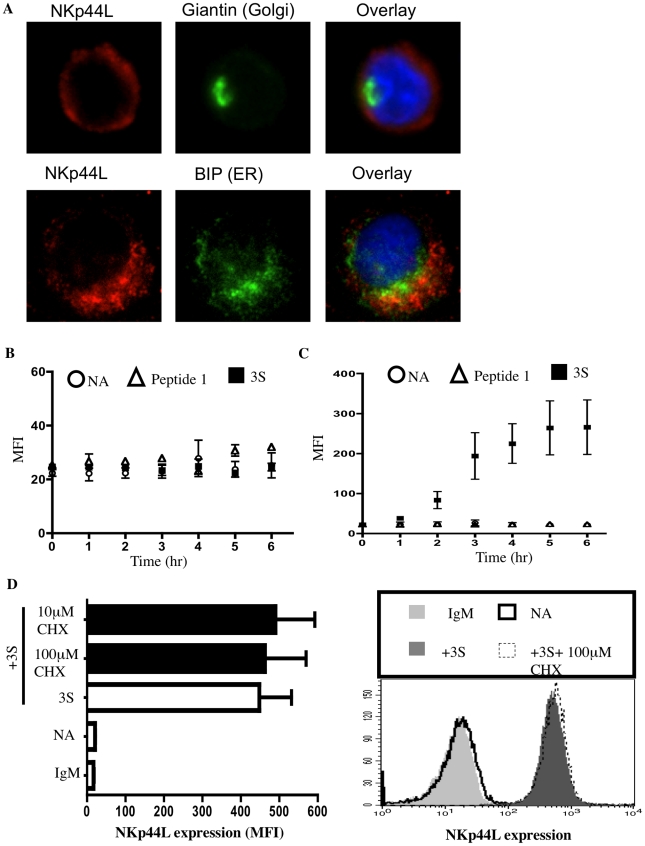
The 3S peptide induces translocation of NKp44L to the cell surface. (A) NKp44L intracellular localization. CD4^+^ T cells were permeabilized and stained for NKp44L (red) and the golgi marker, giantin (green) or the ER marker, GRP78 BiP (green). Nuclei were stained using DAPI (blue). (B) & (C) Kinetic study of total and surface NKp44L expression. CD4^+^ T cells were incubated in absence of peptide (circle), in presence of the 3S peptide (square) or scramble peptide (peptide 1) (triangle) for various length of time (ranging from 0 to 6hrs). Samples were either (B) permeabilized and stained for total NKp44L or (C) stained for surface NKp44L. (D) Cycloheximide does not impede NKp44L surface expression. (Left) CD4^+^ T cells were pre-incubated without inhibitor (open bar) and with 100 µM Cycloheximide (black bars), and then stimulated with the 3S peptide. They were then stained with anti-NKp44L mAb. Unstimulated CD4^+^ T cells stained IgM isotype (IgM) or with anti-NKp44L mAb (NA) were used as negative controls. Means +/− SD of NKp44L MFI from 3 experiments using CD4^+^ T cells from 3 individuals are illustrated. (Right) One representative experiment is illustrated as FACS histograms.

Next, we investigated whether the 3S peptide induces de novo synthesis of NKp44L as well as its translocation to the plasma membrane. High intracellular NKp44L expression was detected by confocal microscopy in purified CD4^+^ T cells in the absence of 3S-peptide stimulation. NKp44L molecules mainly colocalised with the ER marker, GRP78 BiP, within unstimulated CD4^+^ T cells (Figure 3A). Similar results were obtained using the Hela cell line (Supplementary Figure S1). The absence of NKp44L on the surface of CD4^+^ T cells in the absence of 3S-peptide is therefore not due to the absence of NKp44L in the cytoplasm of unstimulated cells but to the inability of these cells to translocate NKp44L to their cell surface. Interestingly, the total level of NKp44L expression remained similar in purified CD4^+^ T cells, without peptide stimulation or treated with the 3S peptide or control peptide (Figure 3B). Concomitantly, at the cell surface, NKp44L steadily and time-dependently increased in purified CD4^+^ T cells after 3S peptide incubation. NKp44L was absent from the surface of untreated CD4^+^ T cells and CD4^+^ T treated with control peptides (Figure 3C). Moreover, cycloheximide treatment did not prevent NKp44L cell-surface induction on primary CD4^+^ T cells by the 3S peptide (Figure 3D). This findings indicate that protein neosynthesis might not be necessary in this signaling pathway and suggests that the 3S peptide directly induces the translocation of pre-existing NKp44L molecules from the cytoplasm to the surface of CD4^+^ T cells.

### The 3S motif of HIV-gp41 triggers Class I PI3K activation

**Figure 4:**
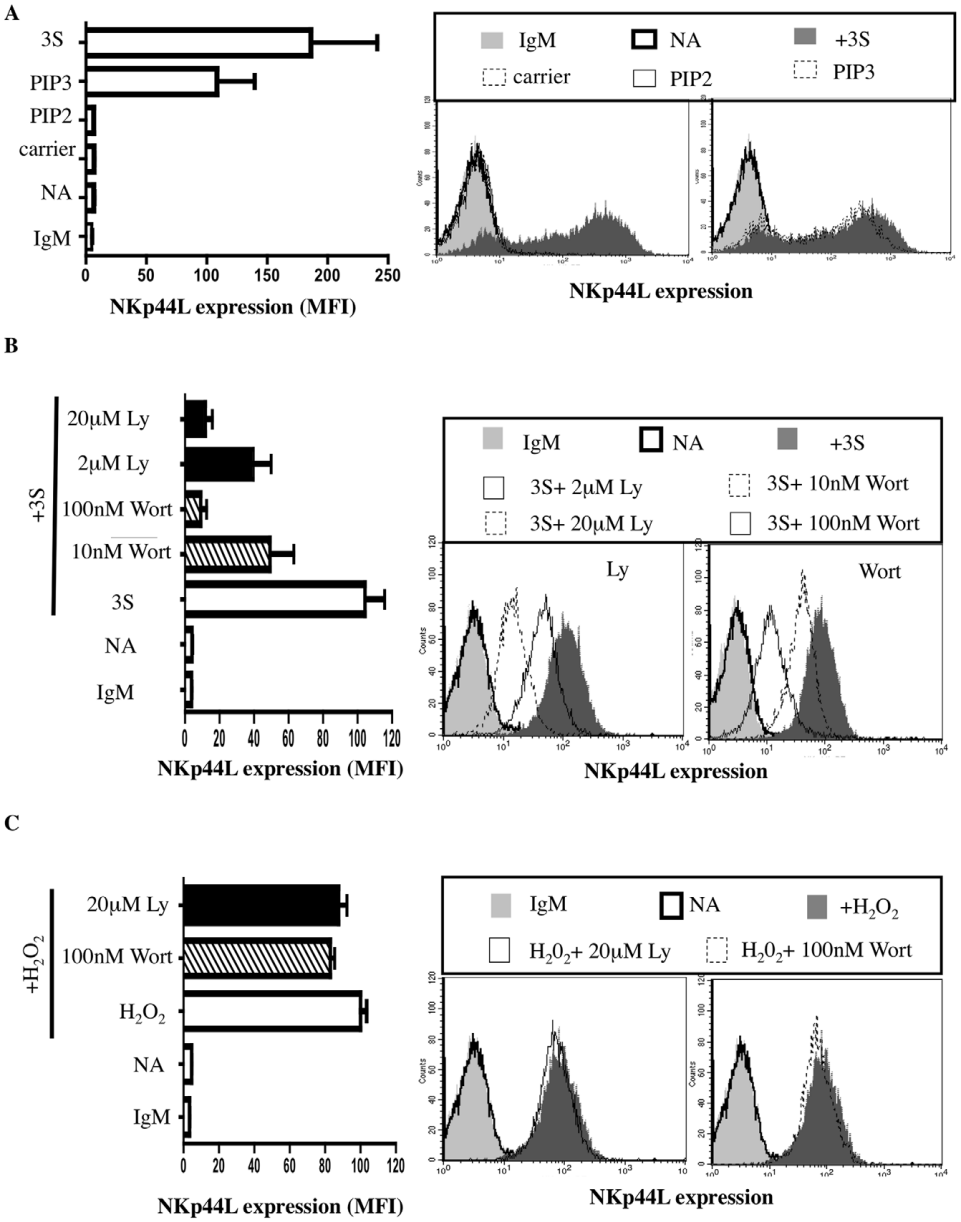
The 3S peptide activates the PI3K. (A) PIP3 induces cell-surface expression of NKp44L. CD4^+^ T cells were stained with IgM isotype control (IgM) or CD4^+^ T cells without stimulation (NA), stimulated with the 5 µg/ml 3S peptide (3S), or 7 µM carrier alone; 7 µM PIP2, or 7 µM PIP3 were then stained with anti-NKp44L mAb. (B) Inhibitors of PI3K block cell-surface expression of NKp44L. CD4^+^ T cells pre-incubated in the absence of inhibitor (Open bar), with 2 or 20 µM Ly294,002 (Black bars), or with 10 or 100 nM wortmannin (Hatched bars) before stimulation with 5 µg/mL 3S peptide were stained with anti-NKp44L mAb. Unstimulated CD4^+^ T cells stained with anti-NKp44L mAb (NA) were used as negative controls. (C) Inhibitors of PI3K do not inhibit cell-surface expression of NKp44L following H2O2 treatment. CD4^+^ T cells pre-incubated in the absence of inhibitor (Open bar), with 20 µM Ly294,002 (Black bars), or with 100 nM wortmannin (Hatched bars), before stimulation with 100 µM H_2_O_2_ were stained with anti-NKp44L mAb. Unstimulated CD4^+^ T cells stained with anti-NKp44L mAb (NA) were used as negative controls. (Left) The mean +/− SD of NKp44L MFI from 3 experiments using CD4^+^ T cells from 3 individuals is illustrated. (Right) One representative experiment is illustrated as FACS histograms.

We examined the signaling mediators upstream of NADPH oxidase activation in further detail. For cytokine signaling, PI3K indirectly activates Rac, one of the regulatory subunits of the NADPH oxidase that leads to H_2_O_2_ production [26]. The role of PI3K in NKp44L translocation was therefore investigated. Purified CD4^+^ T cells were incubated with 7 µM of PIP3, PIP2, or carrier (neomycin) alone. As shown in Figure 4A, PIP3, but neither PIP2 nor carrier alone, was sufficient to induce NKp44L surface expression. Furthermore, similar levels of NKp44L were observed on the surface of CD4^+^ T cells after treatment with either PIP3 or 3S peptide (Figure 4A). Of note, PIP3 did not induce other tested ligands of activating NK receptor, including ULBP1-3, MIC-A/B, CD112 or CD48 (Supplementary Figure S2). To confirm the role of PIP3 production in cell-surface expression of NKp44L, CD4^+^ T cells were pre-incubated, separately, in the presence of two PI3K inhibitors, wortmannin and Ly294,002. These two inhibitors strongly decreased 3S peptide-mediated cell-surface expression of NKp44L, in a dose-dependent manner (Figure 4B). The inability of these two PI3K inhibitors to inhibit H_2_0_2_-mediated NKp44L surface expression demonstrates that NADPH activation occurs downstream of PI3K activation (Figure 4C).

### gC1qR is the receptor of the 3S motif of HIV-1 gp41

Finally, we searched for the receptor of the 3S motif of HIV-1 gp41 at the surface of CD4^+^ T cells. Interestingly, a gp41 epitope, closed to the 3S motif and located in residues 601–620, has been reported to bind to C1q and to activate the complement pathway [16]. In addition, the gp41 epitope located between residues 615–635, which include the 3S motif, binds to soluble factor H, a negative regulator of the complement cascade [18]. We searched the databases for a pleiotropic membrane receptor that is linked to the complement cascade and that activates PI3K and/or NADPH oxidase. Using the above criteria, we identified gC1qR, an atypical receptor of the complement [27], as a potential receptor for the 3S motif of HIV gp41.

**Figure 5:**
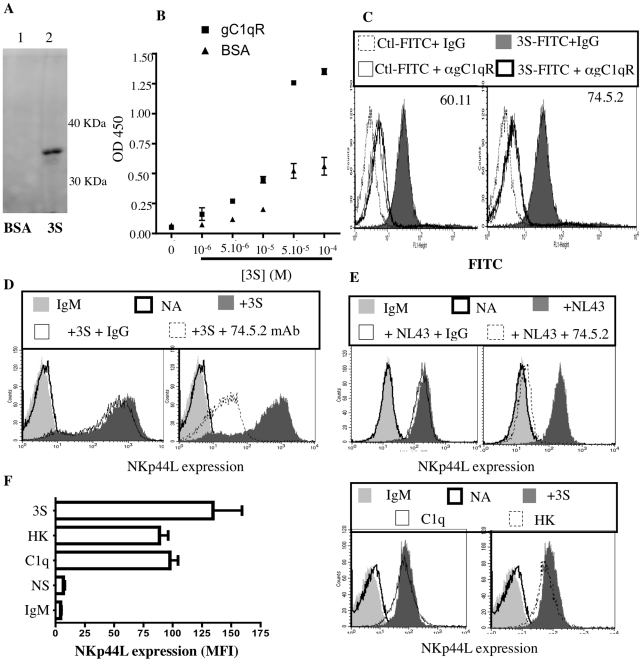
gC1qR is the receptor for the 3S motif of HIV-1 gp41. (A) Immunoprecipitation of gC1qR. Magnetic beads were coated with either BSA or 3S peptide. Coated beads were used to immunoprecipitate the receptor of the 3S motif. Purified proteins were analyzed by Western blot with anti-gC1qR mAbs (74.5.2 and 60.11). Lane **1**: Eluate using BSA coated beads; Lane **2**: Eluate using 3S coated beads. (B) Interaction of gC1qR with 3S peptide by Elisa. Microplate wells were coated with gC1qR or BSA and incubated with increasing concentrations of biotin-conjugated 3S peptide. Biotin was detected with HRP-conjugated streptavidin. (C) Inhibition of 3S interaction by anti-gC1qR mAbs. CD4^+^ T cells were pretreated with 10 µg/ml anti-gC1qR mAb (74.5.2 or 60.11) or IgG1 isotype control, before incubation with 16µg FITC-conjugated 3S peptide. CD4^+^ T cells incubated with 16µg FITC-conjugated scramble peptide (peptide 1) was used as negative control. (D, E) Inhibition of 3S-dependent and HIV-mediated NKp44L stimulation by anti-gC1qR mAbs. CD4^+^ T cells pre-incubated in the absence of antibodies (dark gray), with 10 ug/ml mouse IgG1 (thin solid line), or 10 µg/ml anti-gC1qR 74.5.2 clone (dotted line) before stimulation during 4 hr with (D) 5 µg/mL 3S peptide or (E) wild type HIV virus (NL4,3), were stained with anti-NKp44L mAb. As controls, unstimulated CD4^+^ T cells were stained with IgM isotype control (light gray) or anti-NKp44L antibodies (bold line). (F) Induction of NKp44L surface expression by the natural ligand of gC1qR. CD4^+^ T cells, either unstimulated (NA), or stimulated with 5 µg/ml 3S peptide (3S), 10 µg/ml C1q (C1q) or 10 µg/ml HK (HK), were stained with anti-NKp44L mAb. As control, CD4^+^ T cells were stained with IgM isotype control (IgM). (Left) The mean +/− SD of NKp44L MFI from 3 experiments using CD4^+^ T cells from 3 individuals is illustrated. (Right) One representative experiment is illustrated as FACS histograms.

To determine if gC1qR is the membrane partner of the 3S motif, immunoprecipitation was performed with magnetic beads covalently linked to the 3S peptide. Beads coupled to BSA were used as a negative control. Both sets of beads were then separately incubated with Jurkat CD4^+^ T cells, in the presence of 3,3′–Dithiobis (sulfosuccinimidylpropionate) (DTTSSP), a chemical cross-linker, to link the 3S peptide covalently with interacting proteins. Cells were then lysed, and the interacting proteins eluted from the beads. These proteins were analyzed by both SDS PAGE and Western blotting (with a cocktail of two anti-gC1qR mAbs, 60.11 and 74.5.2). As Figure 5A shows, gC1qR was detected solely in the presence of 3S peptide-coated beads. The specific interaction between the 3S peptide and the gC1qR was confirmed by ELISA. Addition of increasing concentrations of biotin-labeled 3S peptide showed strong and specific binding between gC1qR and the 3S peptide (Figure 5B).

To confirm the role of gC1qR as receptor of the 3S motif of HIV-1 gp41, CD4^+^ T were pretreated with anti-gC1qR mAb (74.5.2 or 60.11) or with the isotype-matched IgG and then stained with FITC-labeled 3S peptide. As Figure 5C shows, the presence of anti-gC1qR mAbs significantly inhibited the binding of FITC-labeled 3S peptide to CD4^+^ T cells. Similar results were observed using biotin-labeled 3S (Supplementary Figure S3A). Next, the ability of anti-gC1qR mAb to inhibit 3S peptide-mediated induction of cell-surface NKp44L expression was tested in purified CD4^+^ T cells. Cells were pretreated with anti-gC1qR mAb (74.5.2) and then incubated with the 3S peptide. As shown in Figure 5D, NKp44L cell-surface expression was strongly inhibited in the presence of anti-gC1qR mAb. Of note, another clone of anti-gC1qR mAb (60.11) was also able to decrease 3S mediated inductions of cell-surface expression of NKp44L but to a lower extend to the 74.5.2 clone (Supplementary Figure S3B). Similarly, anti-gC1qR mAb also inhibited NKp44L surface expression on cells incubated with wild type HIV (NL4.3) for 4hr (Figure 5E).

After demonstrating that the 3S peptide is a new viral ligand of gC1qR, we sought to determine if some endogenous ligands, such as C1q and high molecular weight kininogen (HK), both of which bind to gC1qR, also induce cell-surface expression of NKp44L. Purified CD4^+^ T cells were incubated for 4 h in the presence or absence of C1q, HK, or 3S peptide, and then stained for NKp44L surface expression. As Figure 5F shows, C1q, HK, and the 3S peptide all induced high levels of NKp44L cell-surface expression.

## Discussion

In this report, we demonstrated that the gC1qR protein is the receptor for the 3S motif of HIV-1 gp41 and that their interaction induces NKp44L cell-surface expression on CD4^+^ T cells. A ubiquitous and highly anionic cellular 33-kDa protein, gC1qR was initially identified and characterized as a receptor for the globular heads of C1q (gC1q) [28]. Although it is located mainly in the cytoplasm, particularly in mitochondria, its presence on the surface of diverse human cells has been demonstrated unequivocally, both here and previously [29]. Indeed, we observed here that the 3S peptide interacts with gC1qR expressed on the cell surface of CD4^+^ T cells. Although gC1qR does not have a transmembrane domain, it is thought to transduce signals from the cell surface through interactions with other membrane-bound proteins. For example, the lateral association of gC1qR with β1-integrin is required for C1q-mediated cell adhesion and spreading [30]. Importantly, we showed that two major endogenous ligands of gC1qR, C1q and HK, also induce cell surface expression of NKp44L on CD4^+^ T cells.

Induction of NKp44L surface expression could be relevant for additional bacterial and viral infections. Indeed, gC1qR has been reported to interact with and facilitate entry of numerous microbial and parasitic pathogens, including for example *Listeria monocytogenes*, *Staphylococcus aureus* as well as *Plasmodium falciparum* [31], [32], [33]. More importantly, it is also utilized by some viruses, including HCV, rubella virus, HSV and HTNV [27], [34]. In addition, gC1qR is expressed on several immune cells including macrophages, dendritic and endothelial cells, B and T lymphocytes [27]. The pathogens listed earlier may induce NKp44L surface expression on cells of the immune system mentioned above. Here, we showed that gC1qR is also a receptor for the 3S motif of the HIV-1 gp41 protein. However, *in vitro* the presence of anti-gC1qR mAbs (74,5,2 or 60,11) had no or little impact on the level of infection of purified CD4^+^ T cells by both CCR5- and CXCR4-tropic HIV strains (supplementary Figure S3C). This strongly suggests that these anti-gC1qR mAbs lack the potential to neutralize HIV-1 infection and that gC1qR is not required for HIV-1 infection.

Virus binding to cell-surface receptors may trigger signaling cascades in the host cell. Our examination of the signaling cascade triggered by the 3S motif revealed that PI3K activation plays a critical role in the 3S-mediated signaling that leads to NKp44L translocation to the cell surface. We note that Braun et al previously reported that the binding of internalin B of *Listeria monocytogenes* to gC1qR activates PI3K [31]. In contrast to previous reports of PI3K-mediated activation of Akt [35], Akt phosphorylation was not detected here following 3S peptide stimulation (data not shown). We traced the downstream signaling mediators after 3S-induced PI3K activation. Activated PI3K produced PIP3, which activated NADPH oxidase, probably by promoting GTP-bound Rac. Indeed, one of the Rho GTPase Rac functions that was first characterized in phagocytes was the regulation of the activity of the NADPH oxidase complex, which produces reactive oxygen species (ROS) including H_2_O_2_ [36], [37]. We demonstrated that H_2_O_2_, like the 3S peptide, was able to induce a high level of NKp44L surface expression on CD4^+^ T cells. Remarkably, H_2_O_2_ can also induce surface expression of some ligands for another NK activating receptor, NKG2D, on airway epithelial cells; these ligands include MICA/B and ULBP1-4. In addition, H_2_O_2_ did not modify the intracellular levels of any of these ligands [38]. Similarly, the 3S peptide did not seem to affect intracellular levels of NKp44L. This strongly suggests that H_2_O_2_ is a crucial mediator in the translocation of activating NK ligands to the surface of stressed cells that are flagged for destruction by NK-mediated lysis. This hypothesis is supported by previous reports that H_2_O_2_ also reduces HLA-A, -B, and -C molecules on epithelial cells, probably favoring NK-mediated lysis [38]. Of note, H_2_O_2_ production by the 3S peptide should inactivate PTEN, which would increase PIP3 concentration and therefore H_2_O_2_ production. This suggests a positive feedback loop to amplify NKp44L cell-surface translocation. Similar signal amplification has previously been described for EGF and PDGF stimulations [39].

We also demonstrated that *in vitro* the antioxidant NAC prevented 3S-mediated NKp44L surface expression. Antioxidants have previously been used in clinical trials for treatment of HIV-infected individuals, but with contradictory results. Some have failed to detect any significant beneficial effect on the CD4 cell count of antioxidant supplementation added to HAART treatment, but all showed increased T-lymphocyte proliferation responses [40]. In contrast, other *in vivo* studies have shown that antioxidant supplementation of HIV-infected individuals under HAART improved their CD4^+^ cell count compared with placebo-treated patients [41]. The discrepancies between these results may be due to the type and dose of antioxidant used, as well as to characteristics of the patients.

In the present study, we also showed that stimulation by the 3S peptide activates the p190 RhoGAP-A protein. A previous report showed that Rac-dependent ROS production leads to down-regulation of RhoA through oxidative inactivation of low-molecular-weight protein tyrosine phosphatase and the subsequent activation of p190 RhoGAP-A [22]. In line with that study, our data demonstrated that 3S peptide stimulation promotes the inactive (GDP-bound) form of RhoA. It probably inactivates other Rho family GTPases, notably RhoB, RhoC and TC10, which have all been shown to be inactivated by p190 RhoGAP-A [23], [24]. We note that TC10, a Rho family GTPase mainly localized on vesicular structures, plays a significant role in the exocytosis of GLUT4 and other proteins [42]. More recently, Kawase *et al.* [24] suggested that TC10 may be implicated in three separate steps of exocytosis: loading cargo into secretory vesicles, tethering vesicles to the plasma membrane, and triggering their fusion. Here, we showed that TC10 was necessary for the 3S peptide to induce the translocation of NKp44L to the cell surface. Of note, 3S peptide inactivation of the above Rho family GTPases might, as well as inducing NKp44L surface expression, affect additional signaling pathways and lead to surface expression of other membranes proteins. Indeed, the picture that is emerging today shows that Rho family GTPases transduce signals from pattern recognition receptors and from receptors of antigens, chemokines, and cytokines, as well as adhesion molecules and they function as focal points for crosstalk between different signaling pathways [43].

**Figure 6:**
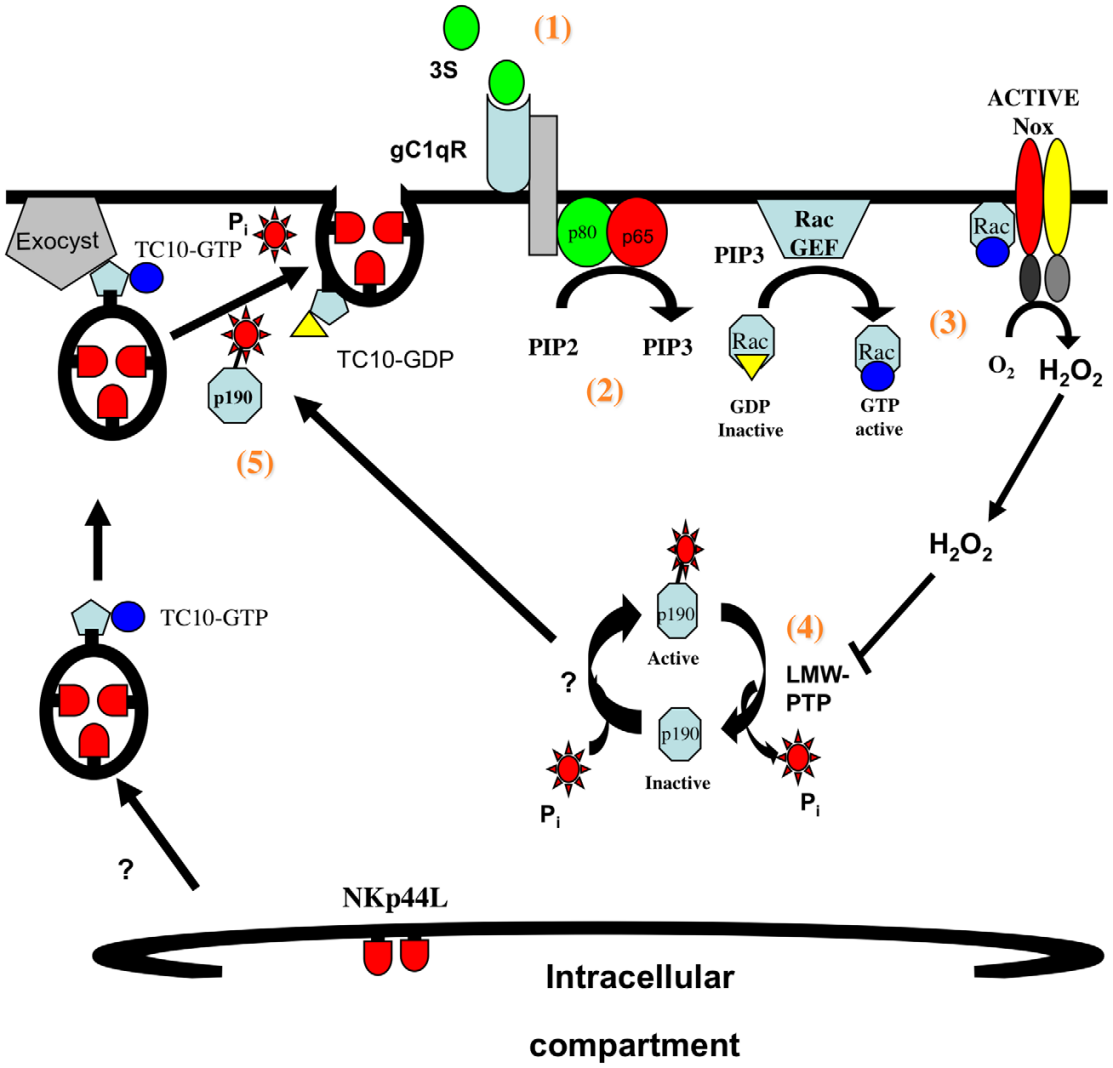
Model of 3S mediated NKp44L translocation on CD4^+^ T cells. 1) The 3S motif of HIV-gp41 binds to its receptor, gC1qR; 2) upon binding gC1qR activates PI3K; 3) activated PI3K indirectly induces H_2_O_2_ production by NADPH-oxidase; 4) H_2_O_2_ produced promotes p190 RhoGAP-A activity; and 5) activated p190 RhoGAP-A then induces GTP hydrolysis by Rho-A and TC10, which are implicated in exocytosis of NKp44L bearing vesicles.

We propose the following model for the translocation of NKp44L to the plasma membrane after stimulation by the 3S peptide (Figure 6): 1) The 3S motif of HIV-gp41 interacts with gC1qR, its specific receptor; 2) this interaction activates PI3K; 3) activated PI3K promotes the activation of the NADPH-oxidase; 4) activated NADPH-oxidase promotes p190 RhoGAP-A activity; and 5) activated p190 RhoGAP-A then induces GTP hydrolysis by Rho-A and TC10, which are implicated in exocytosis. This signaling cascade is similar to that previously described for insulin-induced GLUT4 translocation. GLUT4 surface expression is also dependent on H_2_O_2_ production [44], as well as PI3K [45], and TC10 activation [42].

As we showed previously, the induction of NKp44L surface expression on uninfected bystander cells by the 3S motif renders them highly sensitive to NK lysis, a phenomenon that may lead to the CD4 depletion observed during HIV-1 infection [8], [11]. Interfering with NKp44L expression on the surface of CD4^+^ T cells should therefore be beneficial for HIV-1 infected individuals, by preventing CD4 depletion. Blocking the interaction between the 3S motif and its receptor with gC1qR antagonists should inhibit NKp44L surface expression on CD4^+^ T cells. Moreover, we note that HCV is another pathogen that employs gC1qR to subvert the immune response by inhibiting T cell activation and proliferation [46]. Before gC1qR antagonists can be used to treat HCV or HIV-1 infected individuals, suitable animal models are needed to investigate the *in vivo* effect of gC1qR inhibition in both HCV and HIV-1 infection. Alternatively, HIV-1 infected individuals might be vaccinated with the 3S peptide. A macaque model showed the efficacy of therapeutic immunization with the 3S motif of HIV gp41 against CD4 depletion [15].

## Materials and Methods

### CD4^+^ T cell purification

Leukocytes from whole-blood samples from healthy donors were obtained by buffy coat centrifugation from the hospital blood bank (Hôpital Pitié-Salpêtrière, Paris, France), after approval by the local ethics committee “Etablissement Français du sang”. Written consent from each individual was not required, as blood samples were obtained from healthy anonymous donors. CD4^+^ T cells were purified with CD4 magnetic microbeads (Miltenyi). Flow cytometric analysis demonstrated a purity of >95% CD4^+^ T cells. Purified CD4^+^ T cells were incubated for 3 days with 1 **µ**g/ml PHA-L (Murex) in RPMI-1640 medium supplemented with 10% FCS and then cultured with 100 IU/ml Proleukin 2 (Chiron). The human CD4^+^ T lymphoma Jurkat cell line and the human promonocytic U2 cell line, were both grown in RPMI-1640 medium containing 10% FCS at 37°C and 5% CO_2_. The U2 cell line, which is derived from the promonocytic U937 cell line, was used as it is sensitive to 3S-mediated NKp44L surface induction.

### Reagents

Anti-NKp44L mAb (IgM; #7.1) was previously described [8]. Purified unconjugated and Biotin- or FITC-conjugated 3S peptide from HIV-1 gp41 (NH2-PWNASWSNKSLDDIW-COOH) as well as scramble peptide (NH2-WNWDSKILSDPAWNS-COOH) were purchased from Covalabs (Villeurbanne, France). Complement component C1q (C1q), dibenziodolium chloride (DPI), LY 294,002 hydrochloride, wortmannin, hydrogen peroxide 30% solution, and imidazole were all purchased from SIGMA. Native human high molecular weight kininogen (HK) was purchased from AbD Serotec, PIP3 and PIP2 from TEBU Biosciences, anti-gC1qR (74.5.2 and 60.11), anti-TC10 (T304) and anti GRP78 BiP mAbs from Abcam.

### 2D PAGE and protein identification

For each sample, approximately 2×10^7^ U2 cells were lysed with IEF buffer (7 M urea, 2 M thiourea, 1% CHAPS, 10% isopropanol, 10% isobutanol, 100 mM DTT, 0.5% SB 3–10, 0.2% Bio-Lyte, and 5% triton ×100). Proteins were separated by 2D PAGE. Briefly, protein lysates were mixed with IEF buffer supplemented with 1% IPG pH 3–7 buffer (GE Healthcare), 12 µL/ml Destreak reagent (GE Healthcare) plus bromophenol blue. Samples were added to 18 cm 3–10 NL strips (GE Healthcare) and treated as previously described [47]. SDS-Page was performed on 10% polyacrylamide, and gels were either stained with Imperial Protein Stain (Pierce) or with the PlusOne Silver Staining Kit (GE Healthcare), according to the manufacturer's instructions. Image Master 2D 6.0 software (GE Healthcare) was used to identify proteins significantly modulated from three separate set of experiments. Imperial protein dye was removed from gel pieces with 25 mM ammonium bicarbonate (AMBIC) in 50% ethanol. Gel pieces were dried in acetonitrile (ACN) and then digested with trypsin (PROMEGA). Peptides were extracted twice in TFA 5%, ACN 60%. The combined extracts were dried and resuspended in 0.1% TFA, 50% ACN. Samples were then analyzed by Maldi-ToF (Autoflex by Bruker).

### Measurement of the intracellular H_2_O_2_ concentration

Purified CD4^+^ T cells were pre-incubated with 10 µM DCFH-DA (2′7′ –dichlorofluorescein diacetate) in PBS (without Ca ^2+^ et Mg^2+^) and then incubated in the presence or absence of 20 µg/ml 3S peptide or with 0.2 µg/ml PMA as the positive control. Fluorescence readings were performed every 3 min with a microplate reader (Flexstation 3, Molecular Devices).

### p190 RhoGAP A activity

Purified CD4^+^ T cells were transfected by p190 RhoGAP A wild type (pWT) or RhoGAP A dominant negative (pDN) plasmids, a generous gift from Dr K. Burridge (University of North Carolina, USA), with the Human T cell nucleofector kit (AMAXA) according to the manufacturer's instructions. p190RhoGAP-GFP and p190RhoGAPR1283A-GFP plasmids have been previously described [48]. Transfection efficiency was verified by flow cytometry using GFP fluorescence.

### Rho activity assay

Purified CD4^+^ T cells were incubated in the presence or absence of 5µg/ml 3S peptide for various lengths of time, washed with ice-cold PBS, and then rapidly lysed. For each sample, Rho activity was measured from 12.5 µg total lysate with the G-LISA luminometry-based assay (Tebu-Bio), according to the manufacturer's instructions.

### TC10 knock-down

Purified CD4^+^ T cells were resuspended at 2×10^6^ cells/ml in Accell siRNA delivery media (Dharmacon) supplemented with 100 unit/ml Proleukin 2. Cells were incubated for 72 h with 1 µM siRNA control or with 1 µM Accell siRNA against TC10 (Dharmacon). Two separate siRNAs against TC10 were used independently (5′-CUACUGACCUUGAUGGGUU 3′; 5′- GCAUCAGAUAAUGGUGUUA -3′). Samples were then incubated in the presence or absence of 5 µg/ml 3S peptide before NKp44L staining by flow cytometry. Silencing efficiency of the different siRNAs was first monitored by western blot using anti-TC10 mAb.

### Fluorescence microscopy

CD4^+^ T cells were fixed with 4% paraformaldehyde (PFA) before permeabilisation using 0.1% Triton. Samples were then stained using 1ug anti-NKp44L mAb and 0.75 µg Texas Red conjugated anti mouse IgM mAb. For giantin staining, cells were then incubated with 1/1000 diluted anti giantin mAb and 2 µg diluted Alexa 488 conjugated anti Rabbit IgG. For GRP78 BiP staining, cells were then incubated with 1/1000 diluted anti GRP78 BiP and 2 µg diluted Alexa 488 conjugated anti Rabbit IgG. For TC10 staining, cells were then incubated with 1/100 diluted anti TC10 mAb and 2 µg diluted Alexa 488 conjugated anti Rabbit IgG. Following staining, samples were mounted using ProLong Gold (Invitrogen).

### Flow cytometry

FACS analysis was performed on purified CD4^+^ T cells. Isotype-matched immunoglobulin served as negative controls. For NKp44L surface staining, samples were incubated with 2 µg anti NKp44L antibodies for 1 h at 4°C then incubated in 100 µL of 1∶100 diluted PE anti mouse IgM antibodies (Pharmingen) and CD4-APC antibodies (Beckman Coulter). P190 transfected were stained using only APC anti mouse IgM antibodies (Pharmingen). For total NKp44L level, cells were fixed using BD cellFix solution (BD Biosciences) and permeabilised using 0.5% BSA, 0.1% saponine. Cells were stained using 2 µg purified anti-NKp44L antibodies and 1∶100 diluted PE anti mouse IgM antibodies (Pharmingen). At least 10000 events in the population of interest were detected on a FACScalibur (BD Biosciences). Results were analysed with cellQuest software.

### Delivery of PIP (phospho-inositol) derivatives

PIP derivatives were delivered as previously described [49]. Briefly, solutions containing 250 µM PIP3 or PIP2 in the presence of 250 µM carrier in RPMI medium without serum were freshly prepared in HEPES buffer and then sonicated. Carrier, PIP3, or PIP2 mixtures were added for 4 h to purified CD4^+^ T cells at a final concentration of 7 µM.

### Production, detection, and immunoprecipitation of gC1qR

Recombinant gC1qR protein was purified from *E. coli* transformed with the pET28a-5 plasmid encoding for gC1qR, a kind gift from E. Gouin and P. Cossart (Institut Pasteur, Paris, France) [31]. Briefly, transformed *E. coli* was incubated with 1 mM IPTG for 3 h, and then lysed. The recombinant gC1qR was purified with the TALON dynabeads (Invitrogen), according to the manufacturer's instructions, and eluted in buffer containing 150 mM imidazole. The interaction between the 3S peptide and gC1qR was determined with ELISA after coating with 0.5µg gC1qR, or with BSA as control.

For immunoprecipitation, magnetic beads were coated with 25 µg of 3S peptide, or BSA, as control, per mg of beads, according to the manufacturer's instructions (Ademtech). For each sample, approximately 40×10^6^ cells were mixed with 2 mg of coated beads for 1 h at 4°C, in the presence of 2 mM DTSSP. After incubation, cells were lysed with 1% SDS for 30 min. The magnetic beads were then collected and washed several times with 1 M KCl, followed by 0.1 M NA_2_CO_3_ pH11 and by hypotonic buffer (10 mM Hepes, 10 mM KCl, 1.5 M MgCl_2_) in the presence of protease inhibitor cocktail, as previously described [50]. Purified proteins were eluted, subjected to SDS-PAGE, and then analyzed by Western blot, in the presence of 1.5 µg/ml anti-gC1qR (74.5.2 mAb) and 1 µg/ml anti-gC1qR (60,11 mAb) mAbs. After extensive washing, membranes were analyzed with the Etan Dige Imager.

### Accession numbers

For accession numbers, please see Table 2.

**Table 2:** Accession numbers.

Name	Accession number (Swissprot)	Entry name
TC10	P17081	RHOQ_HUMAN
P190RhoGAP	Q9NRY4	GRLF1_HUMAN
gC1qR	Q07021	C1QBP_HUMAN
C1q (heterotrimer made of)	P02745	C1QA_HUMAN
	P02747	C1QC_HUMAN
	P02746	C1QB_HUMAN
HK	P01042	KNG1_HUMAN

## Supporting Information

Figure S1 NKp44L intracellular localization. Hela cells were permeabilised and stained for NKp44L (red) and the golgi marker, giantin (green) or the ER marker, calnexin (green). Nuclei were stained using DAPI (blue).
(1.00 MB TIF)

Figure S2 PIP3 or H2O2 stimulation does not affect expression of other activating NK ligands including NKG2D ligands. CD4^+^ T cells were stained with isotype control (IgM or IgG2) or CD4^+^ T cells without stimulation (NA), stimulated with the 5 µg/ml 3S peptide (3S), or 7 µM PIP2, or 7 µM PIP3 or 100 µM H_2_O_2_ were then stained with either (A) anti-NKp44L mAb, (B) anti-MIC A/mAb, (C) anti-ULPB1-3 mAbs, (D) CD48 and HLA A, B, C mAbs or (E) CD112 and CD62L mAbs.
(0.86 MB TIF)

Figure S3 Anti-gC1qR mAbs inhibit 3S peptide binding and subsequent NKp44L induction. (A) Inhibition of 3S interaction by anti-gC1qR mAbs. CD4^+^ T cells were pretreated with 10 µg/ml anti-gC1qR mAb (74.5.2 or 60.11 clones) or IgG1 isotype control, before incubation with biotin-conjugated 3S peptide. Peptide was revealed using PE conjugated strepatividin. (B) Inhibition of 3S-dependent NKp44L stimulation by anti-gC1qR mAbs. CD4^+^ T cells pre-incubated in the absence of antibodies (dark gray), with 10 ug/ml mouse IgG1 (thin solid line), or 10 µg/ml anti-gC1qR 74.5.2 clone or 60.11 clone (dotted line) before stimulation with 5 µg/mL 3S peptide, were stained with anti-NKp44L mAb. As controls, unstimulated CD4^+^ T cells were stained with IgM isotype control (light gray) or anti-NKp44L antibodies (bold line). (C) Anti-gC1qR mAbs do not prevent CD4^+^ T cells infection. CD4^+^ T cells were pretreated with 10 µg/ml anti-gC1qR mAb (74.5.2 or 60.11 clones) or IgG1 isotype control or in absence of mAb. Samples were then infected with wild type HIV virus (NL4.3). After 24hrs (left) or 48hrs infection (right), level of infection was monitored by ELISA by dosing p24 antigen.
(0.58 MB TIF)
