# Supplementary figures and images for: HIV gp41 Engages gC1qR on CD4+ T Cells to Induce the Expression of an NK Ligand through the PIP3/H2O2 Pathway
Source: PLoS Pathog. 2010 Jul 1;6(7):e1000975. doi: 10.1371/journal.ppat.1000975 (PMC2895652; doi:10.1371/journal.ppat.1000975)

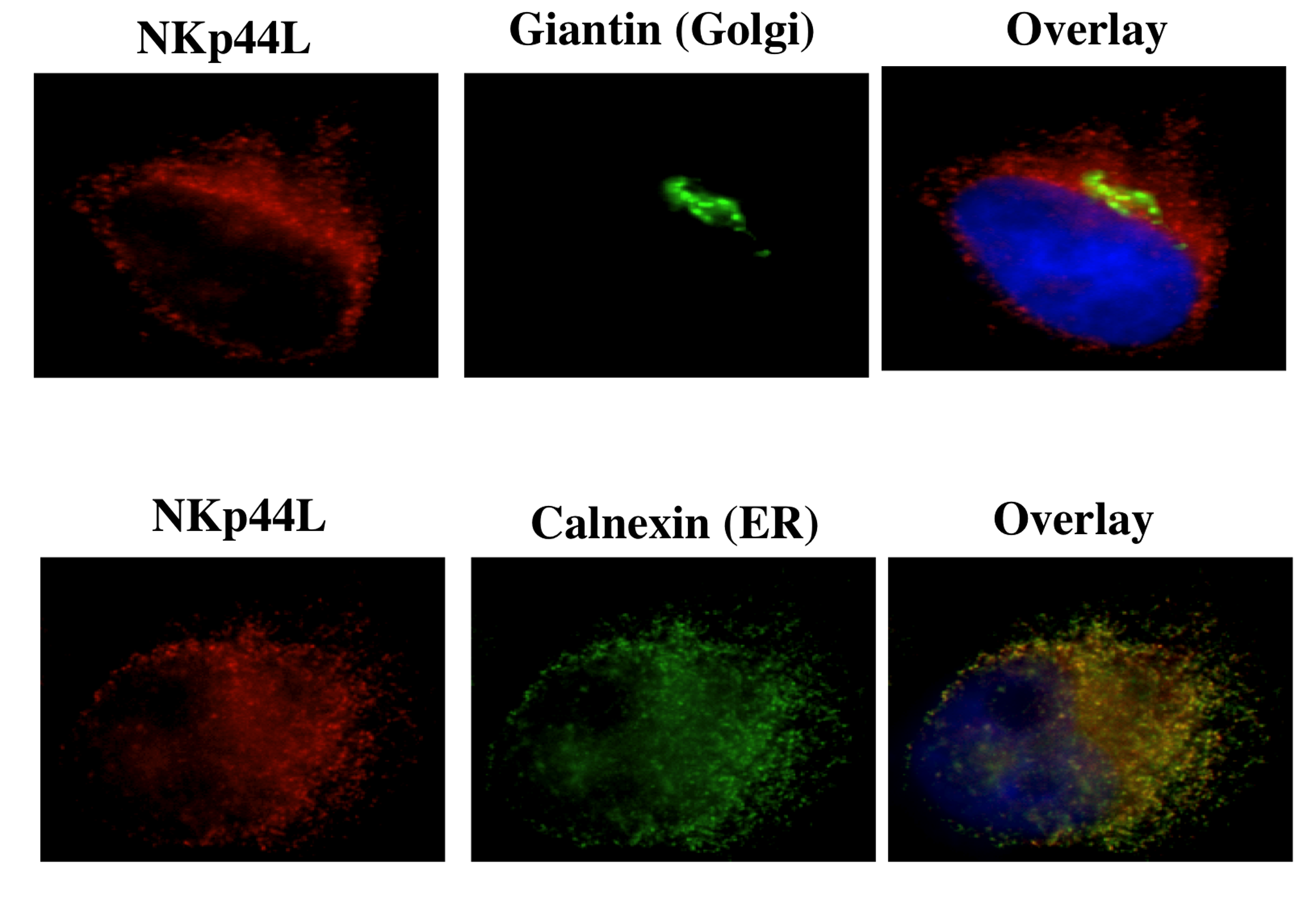

Supplement: Figure S1 — NKp44L intracellular localization. Hela cells were permeabilised and stained for NKp44L (red) and the golgi marker, giantin (green) or the ER marker, calnexin (green). Nuclei were stained using DAPI (blue). (1.00 MB TIF) [file ppat.1000975.s001.tif]

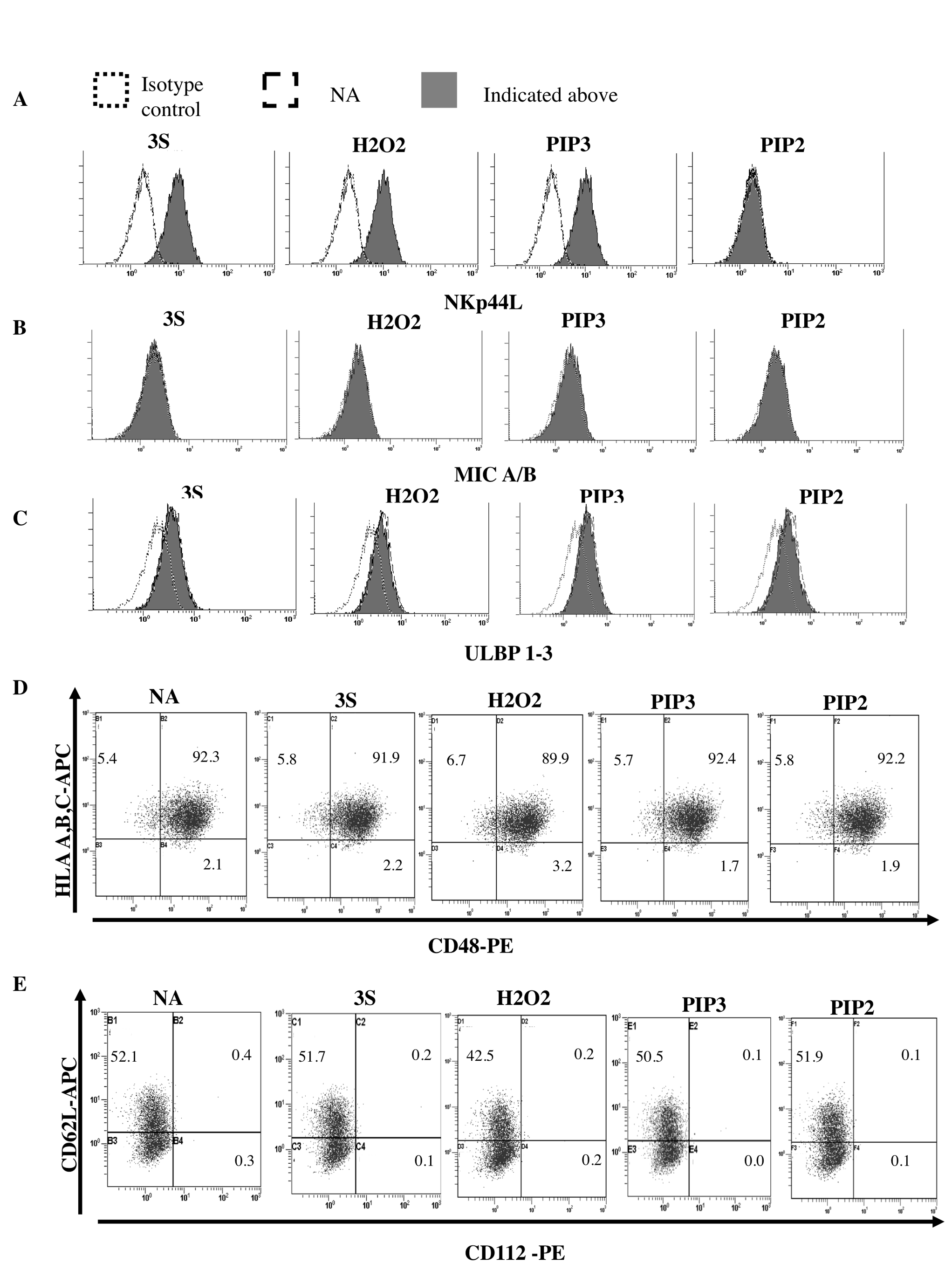

Supplement: Figure S2 — PIP3 or H2O2 stimulation does not affect expression of other activating NK ligands including NKG2D ligands. CD4+ T cells were stained with isotype control (IgM or IgG2) or CD4+ T cells without stimulation (NA), stimulated with the 5 µg/ml 3S peptide (3S), or 7 µM PIP2, or 7 µM PIP3 or 100 µM H2O2 were then stained with either (A) anti-NKp44L mAb, (B) anti-MIC A/mAb, (C) anti-ULPB1-3 mAbs, (D) CD48 and HLA A, B, C mAbs or (E) CD112 and CD62L mAbs. (0.86 MB TIF) [file ppat.1000975.s002.tif]

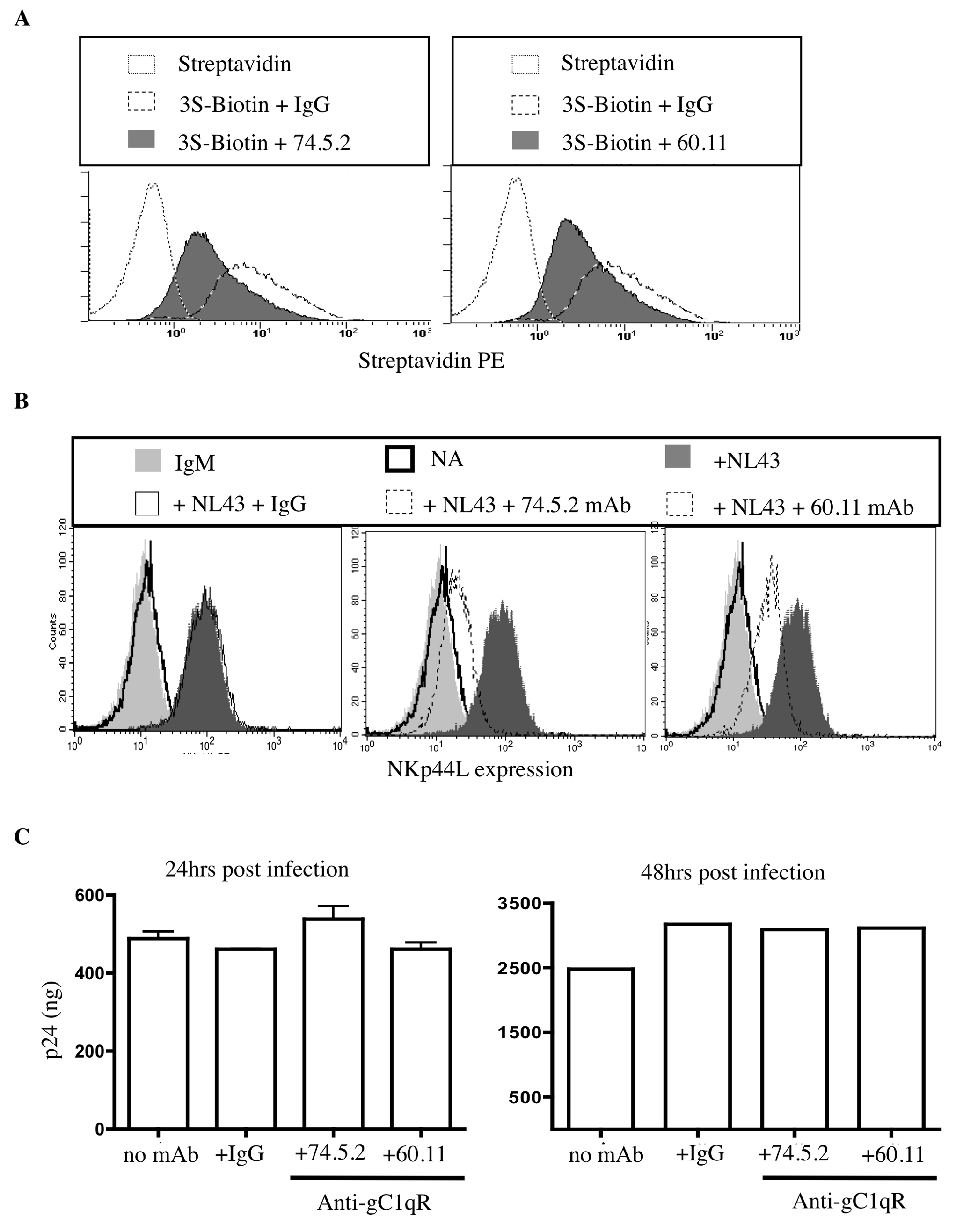

Supplement: Figure S3 — Anti-gC1qR mAbs inhibit 3S peptide binding and subsequent NKp44L induction. (A) Inhibition of 3S interaction by anti-gC1qR mAbs. CD4+ T cells were pretreated with 10 µg/ml anti-gC1qR mAb (74.5.2 or 60.11 clones) or IgG1 isotype control, before incubation with biotin-conjugated 3S peptide. Peptide was revealed using PE conjugated strepatividin. (B) Inhibition of 3S-dependent NKp44L stimulation by anti-gC1qR mAbs. CD4+ T cells pre-incubated in the absence of antibodies (dark gray), with 10 ug/ml mouse IgG1 (thin solid line), or 10 µg/ml anti-gC1qR 74.5.2 clone or 60.11 clone (dotted line) before stimulation with 5 µg/mL 3S peptide, were stained with anti-NKp44L mAb. As controls, unstimulated CD4+ T cells were stained with IgM isotype control (light gray) or anti-NKp44L antibodies (bold line). (C) Anti-gC1qR mAbs do not prevent CD4+ T cells infection. CD4+ T cells were pretreated with 10 µg/ml anti-gC1qR mAb (74.5.2 or 60.11 clones) or IgG1 isotype control or in absence of mAb. Samples were then infected with wild type HIV virus (NL4.3). After 24hrs (left) or 48hrs infection (right), level of infection was monitored by ELISA by dosing p24 antigen. (0.58 MB TIF) [file ppat.1000975.s003.tif]
